# Pandemic (H1N1) 2009 Outbreak at Camp for Children with Hematologic and Oncologic Conditions

**DOI:** 10.3201/eid1701.091499

**Published:** 2011-01

**Authors:** Cori Morrison, Paola Maurtua-Neumann, Myo Thwin Myint, Stacy S. Drury, Rodolfo E. Bégué

**Affiliations:** Author affiliations: Louisiana State University Health Sciences Center, New Orleans, Louisiana, USA (C. Morrison, R.E. Bégué);; Tulane University Medical Center, New Orleans (P. Maurtua-Neumann, M.T. Myint, S.S. Drury)

**Keywords:** Viruses, influenza, children, camp, sickle cell anemia, neoplasms, pandemic (H1N1) 2009, dispatch

## Abstract

An outbreak of influenza A pandemic (H1N1) 2009 occurred among campers and staff at a summer camp attended by children with hematologic and oncologic conditions. The overall attack rate was 36% and was highest among children and adolescents (43%), persons with cancer (48%), and persons with sickle cell disease (82%).

Since it was first identified in April 2009 (*1*), the influenza A pandemic (H1N1) 2009 virus has sickened >1 million persons in the United States (www.cdc.gov/h1n1flu/surveillanceqa.htm). Because of the novelty of this virus, its transmissibility and severity are still under study.

We investigated an outbreak that occurred at a summer camp in northern rural Louisiana. Study approval was provided by the institutional review boards of Louisiana State University Health Sciences Center, Children’s Hospital, and Tulane University Medical Center, New Orleans, Louisiana.

## The Study

The camp opened July 26, 2009 (day 1), with 101 campers and 116 staff. Campers were children with hematologic or oncologic conditions and their nonaffected siblings. All participants were physically examined and questioned so that anyone with presence of or exposure to potentially communicable diseases could be excluded from attending camp. Campers (8–12/patrol) were grouped in age- and gender-specific patrols (B1–B4 and G1–G4) with assigned staff (4–5/patrol); they slept in bunkhouses and had common bathing facilities. All campers and staff dined together and shared various activities.

On day 2, fever developed in a healthy camper in patrol G3 and promptly subsided. Fever also developed in a second camper (patrol B2) with sickle cell disease (SCD). This camper was seen at the local hospital, had a negative rapid influenza test (RIT) result, and was sent home. On day 3, fever and cough developed in 4 children (2 from B2); 1 was tested by RIT with a negative result. Evaluation of the entire B2 patrol found no one else symptomatic. On day 4, fever developed in1 child with cancer (B2) and 1 with SCD (G2 patrol); each had positive test results for influenza A virus.

The number of episodes of fever was considered excessive, and because additional campers reported having fever the camp was closed. At the time of dismissal, all campers and staff were provided masks and instructions about cough etiquette and handwashing; a dose of oseltamivir was also administered, and a prescription for oseltamivir was provided.

Ten days after the camp closed, all attendees were contacted (by email, telephone, and regular mail) to gather information about their outcomes. Clinical signs and symptoms of interest were fever (measured or subjective) or chills; cough or sore throat; muscle pain; and nausea, vomiting, or diarrhea. On the basis of an adaptation of the definition by the Centers for Disease Control and Prevention (www.cdc.gov/h1n1flu/clinicians), persons with fever or chills and symptoms in >2 other categories were classified as having influenza-like illness (ILI); persons with fever alone (without an explanation) and symptoms in 1 other category, or no documented fever but symptoms in >2 other categories, were classified as having probable ILI (P-ILI). A case-patient was defined as a person in whom ILI or P-ILI developed within 10 days of closing of the camp.

Questionnaires were returned by 88 (76%) and 77 (76%) of staff and campers, respectively. Mean age was 22.5 (range 14–69) years for staff and 10.5 (range 5–15) years for campers. Of 88 staff, 56 reported no chronic medical condition; a few reported cancer in remission (n = 7), SCD (n = 3), or other conditions (n = 22: 9 asthma, 2 inflammatory bowel disease, 2 unspecified, and 1 each with epilepsy, gall stones, chronic hepatitis C, immune thrombocytopenic purpura, Kartagener syndrome, mental retardation, porphyria, stroke, and type 1 diabetes mellitus). Of 77 campers, 45 (58%) reported no underlying illness, and a few reported cancer (n = 20: 12 leukemia, 2 lymphoma, 4 solid organ tumor, 2 unspecified; 2 were receiving maintenance therapy, the others had completed treatment), SCD (n = 8), or other (n = 4: 2 asthma, 1 immune thrombocytopenic purpura, and 1 cardiomyopathy).

Of 165 attendees who returned the questionnaire, 59 (38.5%) reported symptoms: fever (40, 68%), cough (30, 51%), sore throat (21, 36%), muscle pain (16, 27%), nausea (10, 17%), diarrhea (9, 15%), vomiting (8, 14%), runny nose (6, 10%), and headache (4, 7%). Abdominal pain, weakness, earache, conjunctivitis, and joint stiffness were rare. Pain crisis developed in 2 patients with SCD. Twenty-five patients met the definition of ILI, and 34 met the definition of P-ILI (Figure). Symptoms were more common among campers than staff (46.8% vs. 26.1%; p = 0.009), persons of younger age (Table 1), and persons with cancer or SCD (Table 2). Proportions were compared with 2-tailed χ^2^ or Fisher exact test by using Epi Info version 3.5 (www.cdc.gov/Epiinfo).

**Figure:**
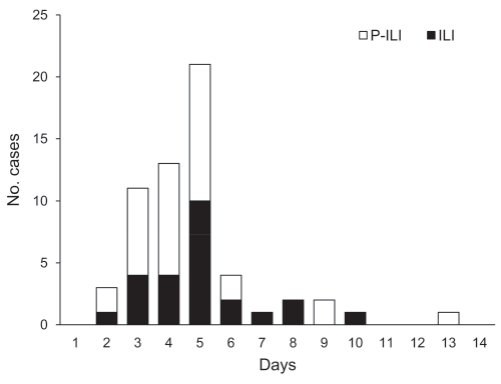
Outbreak curve of influenza-like illness (ILI) and probable ILI (P-ILI) cases during the 4 days of summer camp for children with hematologic and oncologic conditions (1–4) and the 10 days (5–14) after closing the camp, Louisiana, USA, 2009.

**Table 1:** Attack rates of influenza-illness according to age and underlying medical condition at summer camp for children with hematologic and oncologic conditions, Louisiana, USA, 2009*

Age group, y	Underlying condition, no. positive/no. tested (%)		
	No†	Yes‡	All§
5–15	19/54 (35.2)	18/33 (54.5)	37/87 (42.5)
16–30	10/41 (24.4)	11/28 (39.3)	21/69 (30.4)
31–69	0/6 (0)	1/3 (33.3)	1/9 (11.1)
Total	29/101 (28.7)	30/64 (46.9)	59/165 (35.8)

*By χ^2^ test for trend for increasing age groups within each category. †p = 0.049. ‡p = 0.255. §p = 0.027.

**Table 2:** Risk for influenza and hospitalization according to category of underlying condition at summer camp for children with hematologic and oncologic conditions, Louisiana, USA, 2009*

Underlying condition	Risk for influenza symptoms				Risk for hospitalization, if symptomatic		
Staff	Campers	All	Staff	Campers	All
None	10/56 (17.9)	19/45 (42.2)	29/101 (28.7)		0/10/(0)	0/19 (0)	0/29 (0)
Other than cancer or sickle cell disease	7/22 (31.8)	1/4 (25.0)	8/26 (30.8)		0/7 (0)	0/1 (0)	0/8 (0)
Cancer	**4/7 (57.1)**†	9/20 (45.0)	**13/27 (48.1)**†		0/4 (0)	**4/9 (44.4)**‡	**4/13 (30.8)**‡
Sickle cell disease	**2/3 (66.6)**†	**7/8 (87.5)**†	**9/11 (81.8)**‡		**2/2 (100)**†	**6/7 (85.7)**§	**8/9 (88.9)**§
Total	23/88 (26.1)	36/77 (46.8)	59/165 (35.8)		2/23 (8.7)	10/36 (27.8)	12/59 (20.3)

*Values given as no. in category/total no. with condition (%). **Boldface** indicates significance. Data were analyzed by using 2-tailed Fisher exact test for each category compared with persons with no underlying condition. †p = 0.01–0.05. ‡p = 0.0001–0.01. §p<0.0001.

Probably because of small numbers, no difference was evident in the attack rate between staff assigned and unassigned to a patrol, between boy and girl campers, between patrols, or between those who recalled or did not recall being in contact with a case-patient. Twenty-six case-patients sought medical attention, and 12 (2 staff and 10 campers) were hospitalized; 8 had SCD and 4 were in active cancer treatment; all were regular-floor admissions; none required oxygen support or other intensive care; mean length of stay was 4.25 (range 3–6) days; and all recovered uneventfully.

Twenty-five persons (18 campers and 7 staff) were tested for influenza: 13 were tested at an outside facility, of whom 3 had positive results (method of testing unknown); 12 were tested at Children’s Hospital by RIT (BinaxNOW; Invernass Medical, Waltham, MA, USA) and direct immunofluorescence assay (D^3^ Ultra; Diagnostic Hybrids, Athens, OH, USA), and 5 (42%) had positive test results for influenza A virus. Of the 5 positive specimens obtained from patients, 4 were confirmed as pandemic (H1N1) 2009 virus by real-time reverse transcription–PCR.

## Conclusions

Limited preliminary information is available about summer camp outbreaks of pandemic (H1N1) 2009 (*2*). The closest scenarios (school outbreaks) reported attack rates of 3%–33% in the United States (*3**–**5*) and 2%–31% in the United Kingdom (*6**,**7*). Differences between studies and groups probably reflect different levels of exposure and surveillance definitions. In the outbreak reported here, the overall attack rate was 36%; infection was more common among campers (47%), children and adolescents (43%), those with cancer (48%), and those with SCD (82%). Because of their underlying condition, the threshold for evaluation and intervention is lower for children with hematologic or oncologic processes, which might account for some increased reporting of symptoms, hospitalization, and extended stay.

Children with SCD were disproportionately affected; 82% of them reported symptoms, and 89% of those symptomatic were hospitalized because of fever or pain crisis. For seasonal influenza, the hospitalization rate is 56× higher for children with SCD than those without SCD (*8*); the same trend seems true for pandemic (H1N1) 2009 infection. Among those with no underlying condition, the influenza attack rate was lower (28%), few (11%) persons sought medical attention, and none were hospitalized. For persons with no underlying condition, the reported symptoms suggest an illness no more severe than seasonal influenza.

A few limitations should be recognized. First, 25% of attendees did not return the questionnaire. Second, we used a clinical definition of ILI; however, although the limitations of the definition are well recognized, it is a well-accepted tool for outbreak investigation. Third, data are based on recall and report by participants. Fourth, only a few persons were tested to determine the causative agent. However, of the 12 patients we tested at Children’s Hospital, 5 (42%) had influenza A virus and 4 were confirmed as having pandemic (H1N1) 2009 virus infection. This level of positivity is in accordance with sensitivity reported for available tests (*9**,**10*) and suggests that most, if not all, cases of ILI identified were caused by pandemic (H1N1) 2009 virus.

## References

[1] Centers for Disease Control and Prevention. Swine influenza A (H1N1) infection in two children—southern California, March–April 2009. MMWR Morb Mortal Wkly Rep. 2009;58:400–2. 19390508

[2] Centers for Disease Control and Prevention. Oseltamivir-resistant 2009 pandemic influenza A (H1N1) virus infection in two summer campers receiving prophylaxis—North Carolina, 2009. MMWR Morb Mortal Wkly Rep. 2009;58:969–72. 19745803

[3] Iuliano AD, Reed C, Guh A, Desai M, Dee DL, Kutty P, Notes from the field: outbreak of 2009 pandemic influenza A (H1N1) virus at a large public university in Delaware, April–May 2009. Clin Infect Dis. 2009;49:1811–20. 10.1086/649555 19911964

[4] Centers for Disease Control and Prevention. Outbreak of 2009 pandemic influenza A (H1N1) at a school—Hawaii, May 2009. MMWR Morb Mortal Wkly Rep. 2010;58:1440–4. 20057351

[5] France AM, Jackson M, Schrag S, Lynch M, Zimmerman C, Biggerstaff M, Household transmission of 2009 influenza A (H1N1) virus after a school-based outbreak in New York City, April–May 2009. J Infect Dis. 2010;201:984–92. 10.1086/651145 20187740

[6] Health Protection Agency West Midlands H1N1 Investigation Team. Preliminary descriptive epidemiology of a large school outbreak of influenza A (H1N1)v in the West Midlands, United Kingdom, May 2009. Euro Surveill. 2009;14(27):pii=19264. 10.2807/ese.14.27.19264-en 19589329

[7] Smith A, Johnson S, Saldana L, Ihekweazu C, O’Moore E. An outbreak of influenza A (H1N1) in a boarding school in South East England, May–June 2009. Euro Surveill. 2009;14:pii=19263. 10.2807/ese.14.27.19263-en 19589330

[8] Bundy DG, Strouse JJ, Casella JF. Burden of influenza-related hospitalization among children with sickle cell disease. Pediatrics. 2010;125:234–43. 10.1542/peds.2009-1465 20100764 PMC3283164

[9] Centers for Disease Control and Prevention. Evaluation of rapid influenza diagnostic tests for detection of novel influenza A (H1N1) virus—United States, 2009. MMWR Morb Mortal Wkly Rep. 2009;58:826–9. 19661856

[10] Vasoo S, Stevens J, Singh K. Rapid antigen tests for diagnosis of pandemic (swine) influenza A/H1N1. Clin Infect Dis. 2009;49:1090–3. 10.1086/644743 19725784 PMC7107932

